# Choline Catabolism to Glycine Betaine Contributes to *Pseudomonas aeruginosa* Survival during Murine Lung Infection

**DOI:** 10.1371/journal.pone.0056850

**Published:** 2013-02-14

**Authors:** Matthew J. Wargo

**Affiliations:** Department of Microbiology and Molecular Genetics and The Vermont Lung Center, University of Vermont College of Medicine, Burlington, Vermont, United States of America; Institut Pasteur Paris, France

## Abstract

*Pseudomonas aeruginosa* can acquire and metabolize a variety of molecules including choline, an abundant host-derived molecule. In *P. aeruginosa*, choline is oxidized to glycine betaine which can be used as an osmoprotectant, a sole source of carbon and nitrogen, and as an inducer of the virulence factor, hemolytic phospholipase C (PlcH) via the transcriptional regulator GbdR. The primary objective was to determine the contribution of choline conversion to glycine betaine to *P. aeruginosa* survival during mouse lung infection. A secondary objective was to gain insight into the relative contributions of the different roles of glycine betaine to *P. aeruginosa* survival during infection. Using a model of acute murine pneumonia, we determined that deletion of the choline oxidase system (encoded by *betBA*) decreased *P. aeruginosa* survival in the mouse lung. Deletion of the glycine betaine demethylase genes (*gbcA-B*), required for glycine betaine catabolism, did not impact *P. aeruginosa* survival in the lung. Thus, the defect of the *betBA* mutant was not due to a requirement for glycine betaine catabolism or dependence on a downstream metabolite. Deletion of *betBA* decreased the abundance of *plcH* transcript during infection, which suggested a role for PlcH in the *betBA* survival defect. To test the contribution of *plcH* to the *betBA* mutant phenotype a *betBAplcHR* double deletion mutant was generated. The *betBA* and *betBAplcHR* double mutant had a small but significant survival defect compared to the *plcHR* single mutant, suggesting that regulation of *plcH* expression is not the only role for glycine betaine during infection. The conclusion was that choline acquisition and its oxidation to glycine betaine contribute to *P. aeruginosa* survival in the mouse lung. While defective *plcH* induction can explain a portion of the *betBA* mutant phenotype, the exact mechanisms driving the *betBA* mutant survival defect remain unknown.

## Introduction

*Pseudomonas aeruginosa* is an opportunistic Gram negative pathogen that causes a variety of serious and life threatening infections. *P. aeruginosa* lung infection is an important component of disease in people with cystic fibrosis and a substantial source of morbidity and mortality in people undergoing mechanical ventilation [1], [2]. In these settings, antibiotic resistance is a rapidly growing problem, thus there is need for novel therapeutic targets [3], [4]. Acquisition and metabolism of small molecules from the host is an important part of establishment and maintenance of infection [5], [6], [7]. These bacterial pathways define a set of potential – yet largely uncharacterized – targets for drug development. We are interested in understanding the host-derived small molecules that are important for *P. aeruginosa* survival during infection in the mammalian lung.

Analyses of the *P. aeruginosa* transcriptome during growth in cystic fibrosis sputum and normal human mucus showed strong induction of the genes involved in choline transport and catabolism [8], [9], suggesting that choline may be an important molecule for *P. aeruginosa* during infection. Choline is a quaternary amine alcohol that is abundant in the lung, particularly as a moiety on the eukaryotic lipids phosphatidylcholine and sphingomyelin [10], [11]. Nearly all free-living Eubacteria possess a choline oxidation system to generate glycine betaine (GB) from exogenous choline (reviewed in [12]). The near ubiquity of this system suggests an underlying importance to bacterial life, particularly related to osmoprotection and general stress protection [12]. For a variety of bacteria, including well-studied enteric pathogens, the generation of GB for osmoprotection is predicted to be the primary function of choline acquisition and catabolism [13], [14]. For many soil and water-dwelling bacteria, however, there is growing evidence supporting a broader and more varied role for GB.

Unlike the enterics, many common soil and water-dwelling bacteria can aerobically catabolize choline as a sole source of carbon, nitrogen, and energy [15]. In *P. aeruginosa*, choline is oxidized to GB followed by sequential demethylation to dimethylglycine, sarcosine, and glycine [16], [17]. *P. aeruginosa*, like other soil-dwelling Proteobacteria, maintains intracellular GB pools that persist during growth on choline and GB, even in the absence of osmotic stress [17], [18], [19], and *P. aeruginosa* can also maintain an intracellular choline pool [19]. The intracellular pools of both choline and GB are regulated in *P. aeruginosa*, and depletion of either pool alters both growth under osmostress conditions and production of the secreted hemolytic phospholipase C, PlcH [19]. PlcH has been shown to have a role in virulence and bacterial survival in a number of models, including the mouse lung [20], [21], [22], [23], [24], [25], [26]. GB induces the expression of PlcH via the transcription factor GbdR [27], and GbdR regulates the genes involved in GB catabolism [16], directly linking regulation of a secreted virulence factor with metabolism of the host.

The importance of PlcH during infection, and the control of *plcH* transcription by GB activation of GbdR, led us to hypothesize that conversion of host choline to GB would play a role in *P. aeruginosa* biology during lung infection. While we are not the first to propose that catabolism of choline could be an important metabolic process for *P. aeruginosa* during infection [20], [28], [29], the contribution of choline catabolism had not been directly tested in vivo. In this study we have used a mouse model of *P. aeruginosa* lung infection to understand the contribution of choline catabolism to *P. aeruginosa* survival during lung infection. Our data demonstrate that choline catabolism positively contributes to *P. aeruginosa* survival in the mouse lung.

## Materials and Methods

### Ethics Statement

The protocol for animal infection was approved by the University of Vermont Institutional Animal Care and Use Committee, in accordance with Association for Assessment and Accreditation of Laboratory Animal Care guidelines (Animal Welfare Assurance: A3301-01). All procedures were under pentobarbital anesthesia and all efforts were made to minimize animal suffering.

### Strains and growth conditions

*P. aeruginosa* PAO1, *E. coli* (molecular manipulations only), and all derived strains (Table 1) were grown on LB agar or LB broth, and when necessary, supplemented with 50 µg/ml gentamicin for *P. aeruginosa*, and 10 µg/ml gentamicin for *E. coli* (unless otherwise stated). Strains were stored at −80 °C in 20% (v/v) glycerol stocks and freshly streaked plates were used to start the experiments described below.

**Table 1:** Strains and plasmids.

Strain	DB#^1^	Description
P. aeruginosa PAO1-derived strains		
PAO1	MJ79	*P. aeruginosa* wild type [60]
Δ*gbdR*	MJ80	*gbdR* deletion [27]
Δ*plcHR*	MJ143	*plcHR* deletion [26]
Δ*betBA*	MJ311	*betBA* deletion (this study)
*betBA* ^PA14Rev^	MJ518	MJ311 reversion with PA14 *betBA* (this study)
Δ*gbcA-B*	MJ302	*gbcAB* deletion (this study)
Δ*betBA*Δ*plcHR*	MJ437	*plcHR* deletion in MJ311 (this study)
*E. coli* derived strains		
*E. coli* S17/λpir	MJ340	conjugative strain of *E. coli*
*E. coli* S17/λpir	MJ516	carrying pMW166 for *betBA* reversion
*E. coli* S17/λpir	MJ297	carrying pMW122 for *gbcA-B* deletion
Plasmids		
pMQ30		Suicide vector, Gm^R^ [61]
pMW122		PAO1 *gbcA-B* deletion construct (this study)
pMW166		PA14 *betBA* reversion construct (this study)

1 Database designations in the Wargo laboratory. Please use this number for strain requests.

### Creation of deletion strains and complementation constructs

All strains and constructs used in this study are detailed in Table 1. The *betBA* deletion strain in PAO1 was generated using the same primers and methodology as described for the PA14 *betBA* deletion we previously generated [30]. Complementation of Δ*betBA* in trans was confounded by the observation that *betBA* expression is tightly controlled, and over-expression resulted in altered virulence and osmostress phenotypes [19]. In unpublished work, we noted that alteration of *betBA* expression up or down by as little as two-fold impacted *plcH* transcription. We were unable to generate a trans complementation construct that could recapitulate the expression level of the wild-type locus. To address this issue, we regenerated the *betBA* locus in the PAO1 deletion strain using the PA14 *betBA* locus. This region was amplified with primers BetBA-Resc-F (5'-CATGAAGATGCTCAGGGTGA-3') and BetBA-Resc-R (5'-TGTCGGGATAGAGGATGAGG-3') and the resultant product was cloned into the pCR-Blunt vector (Invitrogen), resulting in pMW164. pMW164 was digested with XbaI and HindIII and the ∼5400 bp fragment was ligated into similarly cut pMQ30, resulting in pMW166. Transformation into S17λ/pir, conjugation, and selection of single and double recombinants was done as described previously [30]. Complementation with the PA14 *betBA* copy resulted in a number of defined polymorphisms compared to the PAO1 native locus, one of which removes an AgeI restriction site in the *betB* coding sequence (see www.pseudomonas.com, [31]). This polymorphism could be followed by standard restriction fragment length polymorphism (RFLP) techniques using primers betB-F3 (5'- ATCGAAGGCGAGCAGATTC-3') and betB-R3 (5'-CAGCTCCATGGTCACTTCCT-3'), where these primers generate a 393bp product for both strains that can be cleaved into a 316 and a 77bp set of fragments by AgeI in PAO1, but not in PA14.

We previously reported the Δ*gbcA-B* PAO1 strain, DH841, but due to loss of this stock in our laboratory, we reconstructed the deletion in PAO1 using the same strategy previously employed [16]. The new strain is designated MJ302.

We have previously described the construction of the *plcHR* deletion mutant used for mouse infections [26]. To generate the Δ*betBA*Δ*plcHR* double mutant, we constructed the *plcHR* deletion in the Δ*betBA* (MJ311) strain, using the deletion plasmid described previously [26]. Transformation into S17λ/pir, conjugation, and selection of single and double recombinants was done as described previously [30].

### Mouse lung infection model

We used the oropharyngeal route of mouse lung infection as we previously described [26]. Briefly, cells were streaked onto LB plates from −80 °C stocks. The first plate was restreaked onto a new LB plate after 24 hours and the second plate incubated at 37 °C for 24 hours. Cells from the second plate were used to start 3 ml cultures in LB that were grown for 16–18 hours at 37 °C on a roller drum. From these LB overnight cultures, cells were collected by centrifugation, washed in Dulbecco's PBS (DPBS), and resuspended to give ∼1×10^7^ viable *P. aeruginosa* in 40 µL, with actual inoculum determined by serial dilution of the input suspension. 8–12 weeks old male C57Bl/6J mice (Jackson Labs) were inoculated with 40 µL of the bacterial suspension via oropharyngeal aspiration. Anesthesia, surgery, bronchoalveolar lavage fluid (BALF) collection, organ harvest, and organ homogenization were done as previously described [26] at either 4 or 24 hours post-infection. Viable bacterial counts in organs were determined by serial dilution plating onto PIA followed by incubation at 37°C for 24 hours. White blood cell (WBC) counts in the BALF were done using an Advia automated cell counter (Siemens) and cell type was determined by manual examination of hematoxylin and eosin-stained slides. Cell-free protein content in the BALF was determined by Bradford assay with bovine serum albumin as the standard.

**Figure 1:**
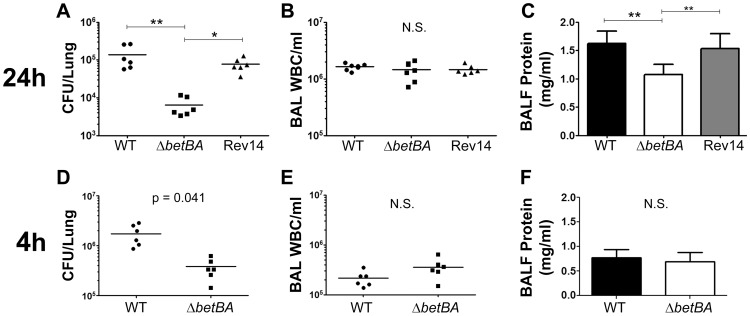
Deletion of the choline catabolic genes reduces *P. aeruginosa* survival in the mouse lung. Mice were infected with *P. aeruginosa* via oropharyngeal aspiration and endpoints measured after 4 or 24 hours. Colony forming units (CFU) per lung, infiltrating white blood cells (WBC), and protein content of cell-free brochoalveolar lavage fluid (BALF) measured after the labeled duration of infection. The data shown are from one representative of three independent experiments. The arithmetic mean is shown for panels with individual mice marked as separate symbols. Error bars in the bar graphs represent standard deviation. Statistical analysis was done using ANOVA followed by Dunnett's Multiple Comparisons test (A–C) or with student's t-test (D–F). Significance in (A–C): * represents p<0.05, ** represents p<0.01. Abbreviations: Rev14 = *betBA* revertant with the PA14 *betBA* locus; N.S.  =  not significant (p>0.05)

**Figure 2:**
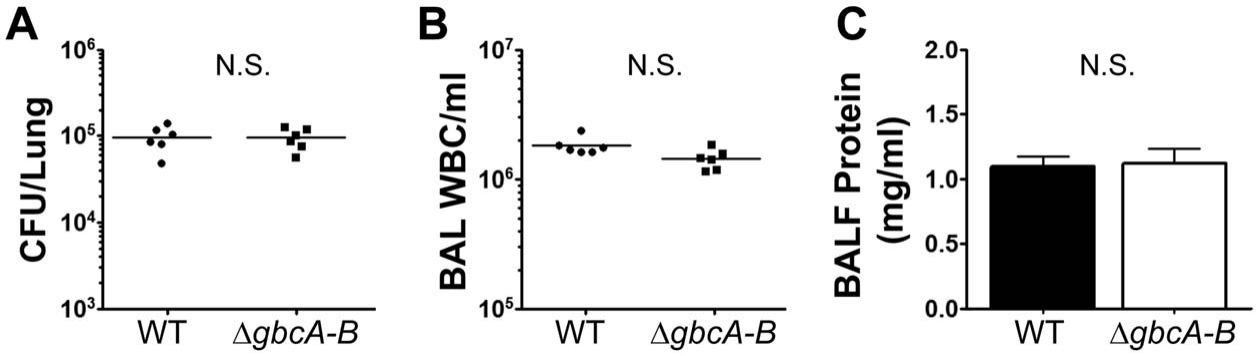
Deletion of GB catabolic genes does not alter *P. aeruginosa* survival in the mouse lung. Mice were infected with *P. aeruginosa* via oropharyngeal aspiration and harvested 24 hours post-infection. The data shown are from one representative of three independent experiments. The arithmetic mean is shown for panels with individual mice marked as separate symbols. Error bars in the bar graph represent standard deviation. Statistical analysis was determined using the student's t-test.

**Figure 3:**
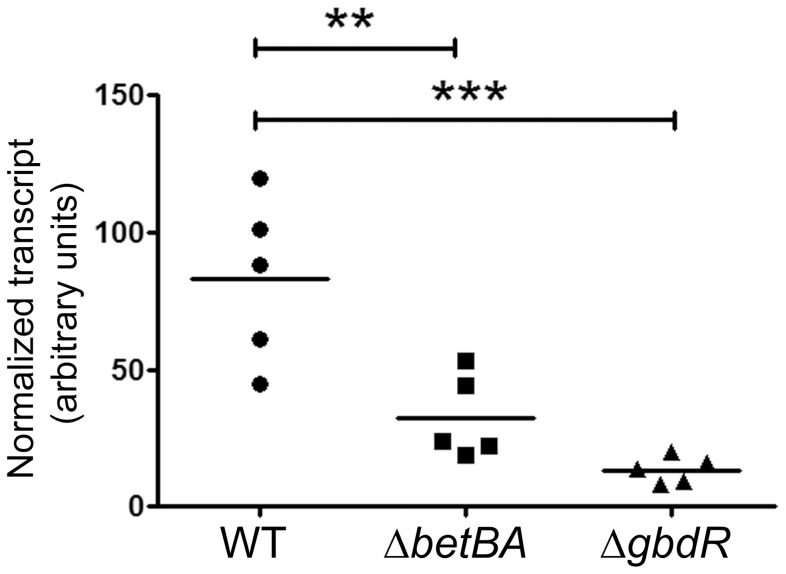
Deletion of *betBA* decreases *plcH* transcript abundance during infection. RNA was isolated from homogenized infected lungs after six hours and subject to quantitative PCR using primers specific for *plcH* normalized to the peptidyl-prolyl isomerase (*ppiD*) transcript. The data shown are from one representative of two independent experiments. The horizontal bar represents the arithmetic mean of each group. Statistical analysis was done using ANOVA followed by Dunnett's Multiple Comparisons test; ** represents p<0.01; *** represents p<0.001. Comparison between the Δ*betBA* and Δ*gbdR* groups was done using ANOVA followed by Tukey-corrected post-test, and these two groups were no statistically different.

**Figure 4:**
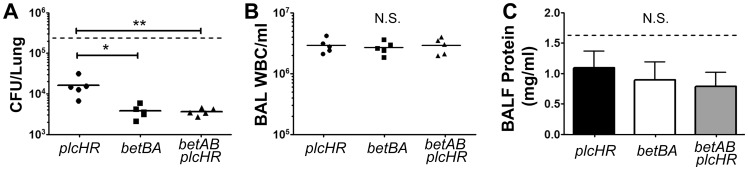
Deletion of *betBA* results in decreased survival compared to deletion of *plcHR*. Mice were infected with *P. aeruginosa* via oropharyngeal aspiration and harvested 24 hours post-infection. The data shown are from one representative of two independent experiments. The arithmetic mean is shown for panels with individual mice marked as separate symbols. Error bars in the bar graph represent standard deviation. Statistical analysis was done using ANOVA followed by Dunnett's Multiple Comparisons test. Significance: * represents p<0.05, ** represents p<0.01, N.S.  =  not significant (p>0.05). The dotted line in Fig 4A and 4C represent the mean of three independent experiments with wild-type PAO1 infection. In Fig. 4B, wild-type is not significantly different from any of the displayed groups.

Mouse experiments for Figures 1 and 2 were performed in triplicate with 5 or 6 mice per group, and in duplicate for Figures 3 and 4. All experiments met the same statistical criteria, i.e. all replicates were consistent with regards to effect size and significance of changes. Inoculation order and harvest order alternated between experiments to eliminate potential issues related to the difference between the duration of inoculation (∼20–30 min) and the duration of harvest (∼1.5 h). For two group comparisons, a two tailed student t-test was used on the data (log transformed for CFU counts). For three group comparisons, data (log transformed for CFU counts) was analyzed by ANOVA followed by Dunnett's Multiple Comparisons test. All calculations were done using GraphPad Prism ®. As described in the Ethics Statement, all animal experiments were reviewed and approved by the University of Vermont Institutional Animal Care and Use Committee.

### qRT-PCR from infected lungs

RNA was extracted from homogenized infected mouse lung as described previously for human sputum [26]. cDNA synthesis was accomplished using Superscript III (Invitrogen) and 5'-NSNSNSNSNS-3' primers in a 20 µL reaction. Quantification of *plcH* and peptidyl-prolyl isomerase (*ppiD*) levels were determined using Taqman duplex PCR to find the ratios of *plcH/ppiD* using the primers and probes previously reported [26]. Statistical analyses were done using ANOVA followed by Dunnett's Multiple Comparisons test and with a Tukey-corrected post-ANOVA analysis to assess statistical differences between the Δ*betBA* and Δ*gbdR* groups. All calculations were done using GraphPad Prism ®.

## Results and Discussion

We are intrigued by bacterial responses to choline as they may mediate important interactions between bacteria and eukaryotes. Choline is abundantly made by eukaryotes and is found at high concentrations in the mammalian lung as a moiety on phosphatidylcholine and sphingomyelin. These two lipids are abundant components of pulmonary surfactant and the outer leaflet of the host cell membranes [10], [11], [32], and are substrates of the hemolytic phospholipase C, PlcH, a known *P. aeruginosa* virulence factor [33], [34]. PlcH hydrolysis of phosphatidylcholine and sphingomyelin generates phosphorylcholine in addition to the respective lipid backbones. Phosporylcholine is hydrolyzed in the periplasm by a specific phosphatase, PchP, which is controlled by GbdR [27], [35], [36]. The resultant choline is then transported into the cytosol by one of at least three transport systems [37], [38], [39]. Once in the cytosol, choline induces expression of the proteins BetA and BetB [40], which sequentially oxidize choline to GB. GB can function as an osmoprotectant, an inducer of *plcH* and other GbdR-controlled transcripts, and as a sole source of carbon, nitrogen, and energy [16], [27], [41]. The multifunctional nature of GB led us to hypothesize that loss of GB acquisition during infection would lead to decreased bacterial survival and/or virulence. To test this hypothesis, we examined the importance of the choline oxidation step, encoded by *betBA*, during infection.

### Deletion of choline catabolic genes reduces *P. aeruginosa* survival in the lung

The abundance of the choline precursors phosphatidylcholine and sphingomyelin in the lung [10], [11] led us and others to predict a role for choline catabolism during infection [16], [28], [29]. To measure the contribution of choline catabolism during *P. aeruginosa* lung infection, we inoculated mice with PAO1 wild-type, a *betBA* deletion strain (Δ*betBA*), or a wild-type reversion strain made by complementing the *betBA* deletion in PAO1 with the PA14 *betBA* genes to reconstruct the *betBA* locus. The *betBA* deletion strain has no defects in colony morphology or growth in LB and we observed no differences in swimming, swarming, twitching, biofilm formation, or pyocyanin production during growth in LB (data not shown). The data shown in Figure 1A demonstrate that deletion of *betBA* results in decreased bacterial survival 24 hours post-infection, which can be complemented by reversion to a functional *betBA* locus (14Rev) (ANOVA p value = 0.005).

The contribution of *betBA* to survival for *E. coli* and *Pseudomonas* has been hypothesized and others have demonstrated in vitro growth defects using genetic or chemical inhibition of *betBA* in host-like (or host-derived) media [14], [42]. To our knowledge, however, this is the first demonstration of a survival defect for a choline catabolic mutant in an animal infection model. The *betBA* locus has not been identified in the high-throughput screens for survival defects in worms, flies, or plants ([25], [43], [44], [45], [46], [47], [48], among others). This may be due to two factors: first, our effect size is not as large as the cutoff used in some of the referenced studies; second, the mammalian lung is particularly rich in choline due to the abundance of pulmonary surfactant, perhaps accentuating the survival defect. This latter factor is further influenced by the relatively low concentrations of carnitine in the lung environment compared to other tissues such as muscle, where carnitine is much more abundant (reviewed in [49], [50]). Because *P. aeruginosa* can synthesize GB from either choline or carnitine [16], [51], [52], we predict that observation of a survival phenotype in other environments may require deletion of both the choline and carnitine catabolic pathways; a genotype not likely during transposon mutagenesis.

### Effects of *P. aeruginosa betBA* deletion on host responses

Deletion of the *betBA* genes decreased *P. aeruginosa* survival in the mouse lung, but we were also interested in the effect of this deletion on the host. Numbers of infiltrating immune cells were not significantly different between the groups (Fig. 1B), nor were the quantity or relative contribution of different infiltrating cell types, which were ∼95% neutrophils in all samples by manual differential counting. However, the protein content of the bronchoalveolar lavage fluid (BALF) – a gross marker of serum leakage and epithelial barrier function – was significantly lower from animals infected by the Δ*betBA* strain (Fig. 1C) (ANOVA p value = 0.0016). A survival difference between the mutant and wild type was detectable at 4 hours post-infection (Fig. 1D), suggesting that a portion of the survival defect is due to events occurring prior to the arrival of the bulk of the circulating neutrophils (Fig. 1E, note scale compared to Fig. 1B). At the 4 hour time point there is no difference in protein content of the cell-free BALF (Fig. 1F).

The host response to *P. aeruginosa* in acute infection models appears largely driven by innate immune response to TLR ligands and the response of the infiltrating neutrophils. Because of this, we suspect that while our bacterial cell numbers are substantially different from the bacteria's point of view, the host is still rapidly clearing infections of both wild-type and the *betBA* deletion mutant (Fig. 1). Most of the responses that give rise to the metrics that we use to grossly assess the host response are likely initiated and propagated normally in both infections. One difference we observed is in the protein content of the BALF (Fig. 1C). Here we suspect the influence of reduced *plcH* expression in the *betBA* mutant, as PlcH has been shown to damage epithelium and endothelium, and we have previously shown that deletion of *plcH* reduces protein concentration in the BALF [26], [34], [53], [54]. We have also measured cytokines typical of *P. aeruginosa* infections in mice (IL-6, TNFα, and KC) and detected no statistically significant change between wild-type and mutant infections at either 4 or 24 hours post-infection (data not shown).

### Loss of GB catabolism does not alter *P. aeruginosa* survival during infection

The data in Fig. 1 confirmed the contribution of choline catabolism to *P. aeruginosa* survival in the mouse lung. As described in the introduction, the product of choline oxidation – GB – has a number of functions in the cell. GB can be used by *P. aeruginosa* as an osmoprotectant, an inducer of transcription via GbdR, and as a sole source of carbon, nitrogen, and energy. In *P. aeruginosa*, GB demethylation is dependent on the *gbcA* and *gbcB* genes [16]. This means that a *gbcA-B* deletion mutant cannot convert GB to DMG and thus is unable to catabolize GB as a sole carbon or nitrogen source [16]. To test the contribution of GB catabolism to virulence, we infected mice with WT PAO1 and an isogenic Δ*gbcA-B* deletion strain and examined experimental endpoints 24 hours post-infection. As shown in Fig. 2A–C, deletion of the *gbcA-B* genes did not alter *P. aeruginosa* survival or host-response in the lung. This demonstrates that GB catabolism is not required for *P. aeruginosa* survival in this model of infection, either as a nutrient source or as a precursor to other metabolites. These data suggest that GB is important for survival in the mammalian lung not through further catabolism (Fig. 2), but for osmoprotection and/or induction of GbdR-dependent transcripts, or a combination of both.

### *betBA* deletion leads to decreased *plcH* transcript during infection

The lack of survival defect in a *gbcA-B* mutant ruled out GB catabolism as the primary contributor to the survival defect of the *betBA* deletion strain (Fig. 2). Two potential alternate reasons for the observed importance of choline conversion to GB are osmoprotection and transcriptional activation of GbdR-regulated genes. Osmoprotection is a biophysical manifestation of the structure of GB and cannot be studied directly using genetics. In addition, there are no robust molecular markers for the low-intensity osmostress predicted to be sensed by *P. aeruginosa* in the host [39]. We have previously reported that deletion of *plcHR*, the genes coding for the phospholipase C virulence factor PlcHR, reduced virulence in our mouse model [26]. If choline was the sole source of GB during infection, we would predict ablation of *plcH* induction in a *betBA* mutant in vivo. Therefore we chose to examine the impact of *betBA* deletion on *plcH* transcript abundance during infection using Taq-man quantitative real-time PCR (qRT-PCR) with the *ppiD* transcript as our control transcript. Expression of *plcH* during infection with wild-type bacteria was variable but high, while these levels were significantly reduced during infection with the *betBA* mutant (Fig. 3) (ANOVA p value = 0.0003). We predict that the *gbdR* mutant infection demonstrates the baseline transcription from the *plcH* locus in the absence of induction (Fig. 3, last group), as our current data supports GbdR as the primary regulator of *plcH* during infection [27].

In a *betBA* mutant, *plcH* transcript levels are reduced during infection, and while the trend was higher expression than in the Δ*gbdR* strain, this difference was not statistically significant (Fig. 3). There are non-choline sources of GB in the host, including free GB and carnitine [14], [55]. Both of these compounds are present at low levels in the lung (low µM quantities) [56]. However, given the transport K_d_'s for both GB and carnitine [38], both could be accumulated during infection. GB could be used directly, while carnitine would need to be catabolized to generate GB, as it cannot induce *plcH* prior to conversion to GB [51].

### Δ*betBA* is epistatic to Δ*plcHR* for the in vivo survival phenotype

The GB generated by BetBA oxidation of choline activates GbdR leading to induction of *plcH* [27]. Therefore, one potential explanation for reduced survival in the *betBA* mutant is reduced *plcH* transcription (Fig. 3). We have previously demonstrated that deletion of *plcHR* reduces *P. aeruginosa* survival in the lung using an infection model identical to our 24 h LB infection [26]. To test the relationship between the Δ*betBA* survival phenotype and that of the Δ*plcHR* strain, we generated a Δ*betBA*Δ*plcHR* double deletion mutant. We compared the *betBAplcHR* double mutant to the *betBA* and *plcHR* single mutants and measured bacterial burden in the lung, infiltrating immune cells, and cell-free protein in the BALF (Fig. 4A–C). For all of these measures, the double mutant was no more or less defective than the single *betBA* mutant. Comparison of the *betBA* single and *betBAplcHR* double mutants to the *plcHR* single mutant demonstrates that loss of *betBA* reduces bacterial survival compared to the *plcHR* mutant (Fig. 4A) (ANOVA p value = 0.0041). This suggests that while *betBA* deletion reduces PlcHR production, there are additional non-*plcHR*-related defect(s) when *betBA* is deleted. These data demonstrate that *betBA* is epistatic to *plcHR* for the survival phenotype.

We show here that deletion of *plcHR* in the background of a *betBA* deletion does not further impact survival of *P. aeruginosa,* but that both the single and double *betBA* mutants survive less well than the *plcHR* single mutant in the mouse lung. We can conclude that the *betBA* mutant has a survival defect that is not solely due to misregulation of *plcHR*. This conclusion is supported by the observation that a *betBA* mutant has poorer survival than the *plcHR* deletion (Fig. 4A), yet still induces *plcHR* in the lung, albeit at a lower level than wild type (Fig. 3). A likely mechanism for the decreased survival in the *betBA* mutant compared to Δ*plcHR* is that GB is important for one of its other roles in *P. aeruginosa*, two candidates being stress protection and induction of non-*plcH* transcripts via GbdR. For some intracellular bacteria, choline, GB, or carnitine transport is important for survival in phagocytic cells [57], [58], [59]. If the killing efficiency of wild-type *P. aeruginosa* by phagocytes is not 100%, any defect in the ability to escape sub-optimal phagocytosis would manifest as a survival defect. This could be explored in vitro using macrophages and neutrophils from mouse strains defective for antimicrobial activities, such as NO production, defensins, cathelicidins, and complement proteins. While PlcH is the only known secreted virulence factor controlled by GbdR, GbdR controls a regulon that includes genes of unknown function (Hampel and Wargo, unpublished data); therefore one of these genes may be important for infection. GB may also be performing some unknown function in the cell, whether through alternate transcription factors or otherwise altering the biology of the cell. Finally, it remains a formal possibility that BetB, BetA, or both, perform additional functions in the cell in addition to their role in choline catabolism.

### Conclusions

Conversion of choline to GB contributes to *P. aeruginosa* survival during infection. This finding answers our primary objective and supplies data in support of longstanding hypotheses in this field [28], [29]. While loss of GB catabolism does not alter survival in the mouse lung, the mechanism behind the survival defect of the *betBA* mutant remains unknown. We are interested in unraveling the mechanistic contributions of PlcH and GB accumulation to the Δ*betBA* survival defect. Given the evolutionary conservation of choline oxidation to GB and the apparent prevalence of a stable GB pool in many environmental bacterial species [19], we predict that GB accumulation will be important for survival in other Gram negative opportunistic pathogens.
